# A Comparative Study on Nutritional Status and Body Composition of Urban and Rural Schoolchildren from Brandsen District (Argentina)

**DOI:** 10.1371/journal.pone.0052792

**Published:** 2013-01-07

**Authors:** Maria Florencia Cesani, Mariela Garraza, María Laura Bergel Sanchís, María Antonia Luis, María Fernanda Torres, Fabián Aníbal Quintero, Evelia Edith Oyhenart

**Affiliations:** 1 Instituto de Genética Veterinaria. Facultad de Ciencias Veterinarias, Universidad Nacional de La Plata, UNLP-CONICET CCT La Plata, La Plata, Provincia de Buenos Aires, Argentina; 2 Facultad de Ciencias Naturales y Museo (UNLP), La Plata, Provincia de Buenos Aires, Argentina; 3 Facultad de Filosofía y Letras, Universidad de Buenos Aires (UBA), Ciudad Autónoma de Buenos Aires, Argentina; Charité University Medicine Berlin, Germany

## Abstract

The purpose of this study was to analyze whether nutritional status and body composition varies according to the environment of residence (urban or rural) of children in the Brandsen district (Argentina). Weight, height, arm circumference and tricipital and subscapular skinfolds were performed in 1368 schoolchildren aged 3 to 14. NHANES III reference was used to estimate nutritional status -underweight, stunting, wasting, overweight, and obesity- and to evaluate body composition -deficit and excess of adipose (DA, EA) and muscular (DM, EM) tissues of the arm-. Central fat distribution (CFD) was estimated using the subscapular-tricipital index. A structured questionnaire was implemented to evaluate socio-environmental characteristics. Nutritional categories based on body size and body composition were compared between urban and rural areas of residence using Chi-squared tests (χ2). The results indicated for the total sample: 1.1% underweight, 6.9% stunting, 0.4% wasting, 12.1% overweight, 9.7% obesity, 22.0% DM, 2.5% EM, 0.1% DA, 17.6% EA, and 8.5% CFD. Significant differences between urban and rural areas were found only for CFD. The socio-environmental analysis showed that while access to public services and housing quality was significantly better in the urban area, a considerable number of city households lived under deficient conditions, lacked health insurance and had low socioeconomic level. Fifty-three percent of the undernourished children had DM without urban-rural significant differences, and none of them showed DA. In the overweight plus obesity group, 62.8% presented EA, 6.4% EM, 4.7% DM, and 22.8% CFD. The highest percentages of DM and CFD were recorded in rural areas (p = 0.00). We conclude that the child population shows the “double burden” of malnutrition. The environment of residence does not promote any differentiation in the nutritional status. Nevertheless, the increment of central adiposity and, in some cases of muscle deficit in rural children, suggests a consumption of unbalanced diet.

## Introduction

A considerable body of literature has documented the rural-urban health disparity in children and adults from developing countries. Most of these studies were focused on discrepancies in child nutritional status, and demonstrated that, on average, urban children are less likely to suffer stunting and underweight [1]–[2]–[3]–[4]–[5]–[6]–[7]. Nevertheless, the urban advantage has been fading in the last decades, since the fast changes in the diet and lifestyle of city dwellers -resulting from industrialization, urbanization and globalization-, have triggered marked consequences on the health and nutritional status of the populations [6]. Urban diets include a large consumption of fat-rich food, more sugar, and more processed foods [8] and the accompanying lifestyle is less physically active [9]–[10]. The types of jobs available in urban areas are often more sedentary than those in rural areas, causing changes in physical activity levels. Likewise, changes in leisure-time activities and the different types of transportation available (e.g. buses, cars) result in more sedentary lifestyles [11]–[12]. As a result, an increasing amount of overweight and obese people has been recorded in urban areas throughout the world.

Moreover, there is growing recognition of the emergence of a “double burden” of malnutrition, with under- and over-nutrition occurring simultaneously among different population groups in developing countries [13]–[14]–[15]–[16]. Accordingly, in Argentina undernourishment and overweight have been reported to coexist in many populations [17]–[18]–[19]–[20]–[21]–[22]. In addition, it was noted that overweight and obese children could simultaneously present excess of fat tissue and deficit of muscle when they live under impoverished residence conditions [23]. Such results illustrate the complexity of this phenomenon and highlight the importance of studying body composition as an essential complement to studies of nutritional assessment [24].

Since 2005, our research team has been working in the Brandsen district (Argentina) analyzing nutritional status in the preschool children population [25]–[26]–[27]. We have analyzed urban- rural differences and reported the coexistence of undernutrition, overweight and obesity [19]. Even so, while undernutrition was prevalent in suburban areas where the worst social and environmental conditions for optimal growth of children were observed, the highest rates of obesity were recorded in rural areas [19]. Nevertheless, we have not yet analyzed older children or compared body composition between urban and rural areas. Therefore, the aim of this paper was to analyze whether nutritional status and body composition varies according to the environment of residence (urban or rural) of children in the Brandsen district.

## Materials and Methods

### Ethics statement

This study was approved by the University of La Plata (UNLP), local authorities, and educational and sanitary staff of Brandsen district. Research protocols followed the principles outlined in the Helsinki Declaration and successive modifications as well as those under the National Law N° 25.326 on the protection of personal data.

The study's goals and procedures were explained during meetings held in each school. Informed consent was signed by the children's parents. Children whose parents did not sign the forms were not measured. In addition, the children themselves were consulted and only those who agreed (orally) were included in the study.

### Population

**Figure 1:**
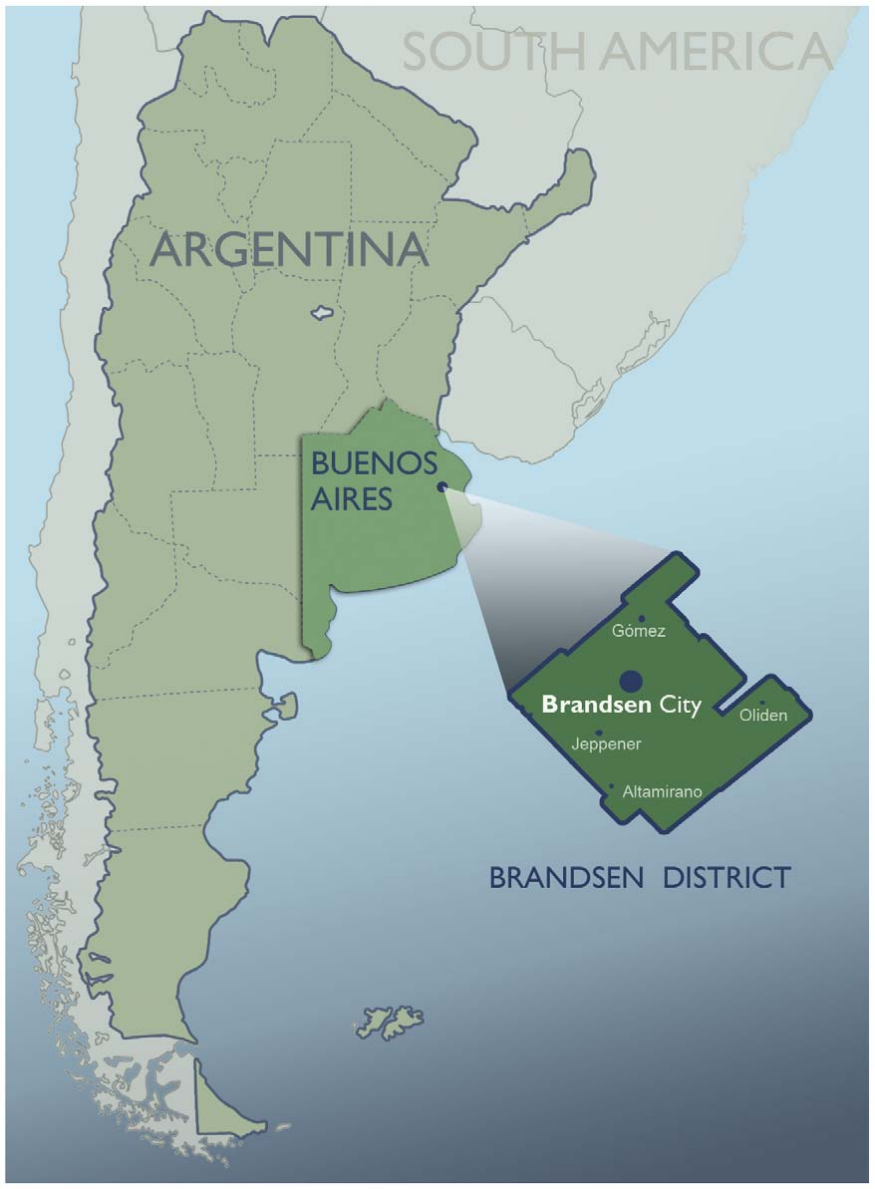
Geographic location of Brandsen District (Buenos Aires, Argentina).

Brandsen is a district located in the northern area of Buenos Aires Province; its major city is Coronel Brandsen which is situated just a few kilometers away from the cities of La Plata and Buenos Aires (35°10′S, 58°13′W) (Figure 1). According to the last Argentine National Census (2010), the maximum total population was 26.352 inhabitants, with 85% concentrated in urban areas and the other 15% either distributed in smaller towns (fewer than 2,000 inhabitants) or spread in rural areas [28]. Although both agriculture and agro industry contribute to regional economy, it is the third sector which participates with a greater percentage of Gross Domestic Product (GDP) [28]–[29].

### Sample

A cross-sectional anthropometric study was performed in 1368 children (48% boys 52% girls), aged 3 to 14 years, attending urban (Coronel Brandsen town) and rural (Jeppener, Altamirano, Gómez, and Oliden villages) schools of Brandsen district (Table 1). We employed the criteria suggested by the Instituto de Estadísticas y Censos de Argentina (INDEC) to determine whether a population was urban or rural. Their criterion considers as “urban” any population with more than 2000 inhabitants [29].

**Table 1:** Sample composition.

Age (years)	N	Male	Female	Urban Population	Rural Population
3–3.9	170	85	85	136	34
4–4.9	162	83	79	116	46
5–5.9	150	77	73	98	52
6–6.9	118	60	58	81	37
7–7.9	124	64	60	97	27
8–8.9	87	37	50	72	15
9–9.9	123	51	72	83	40
10–10.9	125	51	74	96	29
11–11.9	106	53	53	66	40
12–12.9	96	48	48	65	31
13–13.9	66	22	44	39	27
14–14.9	41	20	21	21	20
Total	1368	651	717	970	398

Data were collected from 20 public educational institutions (kindergarten and elementary school), representing 70% of the total schools of the district. Children with chronic diseases or pathological conditions existing at the moment of the study were excluded.

### Anthropometric study

The anthropometric study was carried out following standardized protocols [30].

The following variables were recorded: age: obtained from the identification cards or from the school's records; body weight (Kg): measured on a digital scale (accuracy, 100 g) with the subjects lightly clothed (the weight of the clothes being subtracted); height (cm): using a portable vertical anthropometer (accuracy, 1 mm); arm circumference (AC): (cm) using an inextensible tape measure (accuracy, 1 mm); tricipital and subscapular skinfolds (TF and SF) (mm): with a Lange caliper using constant pressure (accuracy, 1 mm). Body-mass index (BMI) was calculated as weight (kg) divided by squared height (m^2^).

To estimate nutritional status, the NHANES III reference was used. The cut-off value was <5th percentile to determine low weight-for-age (underweight), low height-for-age (stunting), and low weight-for-height (wasting). Individuals were classified as overweight or obese when their BMI was in the 85th through 95th percentile or above the 95th percentile, respectively [31].

Body composition was estimated on the basis of total (TA), muscle (MA) and fat (FA) areas following Frisancho [31].
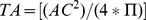


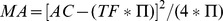


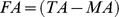



A cut-off point of <5th percentile and >95th was used to determine deficit and excess of adipose (DA, EA) and muscular (DM, EM) tissues of the arm. Finally, fat distribution (central or peripheral) was evaluated using the subscapular-tricipital index (STI) calculated as the ratio between subscapular and tricipital skinfolds (STI = 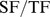) [32]. A STI score higher than 1 was considered as an indicator of high risk of central fat distribution (CFD) [33].

### Socio-environmental study

A structured questionnaire, completed by the parents, was implemented to evaluate socio-environmental characteristics, and to measure housing variables by means of information regarding structural and physical amenities [27]. These characteristics provided information about interior and exterior housing conditions.

We asked about: building materials (type of materials used in their construction: low-quality prefab, fired-brick masonry, makeshift materials, and so forth); source of drinking water (piped water system, protected well, rain-tank storage, or unprotected well); wastewater disposal (sewage system or septic tanks (cesspool)); fuel for cooking and heating (piped gas, bottled gas (cylinder), kerosene, or firewood); pavement; electricity, waste collection, and critical crowding (more than three persons per room).

Regarding socio-economic status (SES), the following variables were considered: lodging or home-tenure status (house owner, lease holder, or free lodging); parental education (elementary, high school, university); parental job (employed or formal worker, unskilled worker or unqualified worker who performs mostly temporary jobs, informal worker or without work contract, autonomous worker or freelance jobs, and unemployed); health insurance (medical insurance at the expense of the employer or paid by the person -fee for service health insurance plans-); public assistance (referring to national or local programs from government agencies, NGOs, or other entities, that benefit poor families by supplementing their food budget -nutritional support- and/or by providing cash relief to the heads of households -monetary support-; farming (animal husbandry, orchard, or horticulture).

### Statistical analysis

Nutritional categories based on body size and body composition were compared between urban and rural areas of residence using Chi-squared tests (χ2). All statistical procedures were made with SPSS 12.0 statistical program.

## Results

Table 2 shows the results of the socio-environmental analysis. Although most of the urban and rural houses were built with fired-brick masonry, the quality of building materials was lower in the rural area. With the exception of electricity, urban households had better access to public services (pavement, piped water system, waste collection and sewage system). In regards to the fuel for cooking and heating, less than 30% of the families of the district had piped gas. Thus, bottled gas (cylinder) was used as main fuel. Additionally, in the rural area a large amount of families used firewood as an alternative source of fuel.

**Table 2:** Socio-environmental analysis.

	Percentages (%)			
	Urban	Rural	χ*2*	*p*
**HOUSING VARIABLES**				
**Building materials**			12.62	0.006
Fired-brick masonry	86.6	77.7		
Low-quality prefab	6.6	8.6		
Makeshift materials	3.6	8.2		
Others materials	3.2	5.5		
**Availability of public services** *				
Pavement	33.7	26.2	4.53	0.033
Piped water system	57.9	46.7	8.88	0.003
Electricity	92.9	92.1	0.15	0.700
Waste collection	81.2	63.3	31.76	0.000
Wastewater disposal (sewage system)	27.8	11.4	26.01	0.000
**Fuel for cooking and heating** *				
Piped gas	21.7	28.4	4.39	0.036
Bottled gas (cylinder)	75.9	69.4	3.89	0.031
Firewood	29.7	47.0	23.89	0.000
**Critical Crowding** *	49.4	45.5	0.99	0.320
**SOCIOECONOMIC STATUS VARIABLES**				
**Lodging or home-tenure status**			21.83	0.000
House owner	70.4	54.4		
Lease holder	11.6	14.6		
Free lodging	18.0	31.0		
**Parental Education**				
**Father**			6.71	0.010
High school and University	63.9	52.8		
Elementary school	6.8	2.3		
No Data	29.6	45.0		
**Mother**			9.24	0.002
High school and University	64.0	51.8		
Elementary school	10.9	3.8		
No Data	25.1	45.0		
**Parental Job**				
**Father**			0.72	0.697
Formal and Free Lance Job	51.3	38.7		
Unqualified and Informal Job	14.6	12.1		
Unemployed	3.9	3.8		
No Data	30.1	45.5		
**Mother**			18.62	0.000
Formal and Free Lance Job	30.0	16.1		
Unqualified and Informal Job	2.2	4.8		
Unemployed	34.8	25.1		
No Data	33.0	54.0		
**Health insurance** *	48.8	48.5	0.01	0.927
**Nutritional support** *	22.5	14.4	7.03	0.008
**Monetary support** *	16.7	13.5	1.32	0.251
**Animal husbandry** *	7.8	26.2	56.52	0.000
**Orchard or horticulture** *	5.7	19.2	40.31	0.000

* The percentage indicate the presence of the variable.

Critical crowding was high in both urban and rural populations, reaching percentages of 49.4 and 45.5 respectively. On the other hand, most of the families owned their homes, but this type of accommodation was more frequent in the urban area, while in the rural areas, 31% of households had free hosting.

Regarding parental education, city-dwellers accredited higher educational levels. Similarly, a greater percentage of parents with formal or free lance jobs were recorded in the urban area. However, the differences between urban and rural areas were only statistically significant for the mothers.

The amount of families with health insurance and monetary support was similar in urban and rural areas. However, city-dwellers received more nutritional support from government agencies, NGOs or other entities, than those in rural areas. Conversely, orchard farming and animal husbandry for consumption were more frequent among rural residents.

The analysis of nutritional status indicated that 1.1% of the total sample (urban plus rural population) suffered underweight, 6.9% stunting, 0.4% wasting, 12.1% overweight, and 9.7% obesity (Table 3). Differences between boys and girls were observed only for stunting (*X^2^:*5.02, p: 0.025) and obesity (*X^2^:*50.56; p: 0.033) at the age 10.0–10.9. Therefore, data for both sexes were pooled for subsequent statistical analyses.

**Table 3:** Differences in prevalence of underweight, stunting, wasting, overweight and obesity between urban and rural areas.

Nutritional Status	Prevalence (%)				
	Total	Urban	Rural	*X* ^2^	*p*
Underweight	1.1	0.9	1.5	0.87	0.350
Stunting	6.9	6.4	8.3	1.58	0.209
Wasting	0.4	0.3	0.5	0.29	0.591
Overweight	12.1	11.9	12.6	0.13	0.715
Obesity	9.7	9.7	9.8	0.00	0.951

Prevalence of undernutrition, overweight and obesity was similar in both urban and rural areas (Table 3).

The results of the body composition analysis are shown in Table 4. Twenty-two percent of the children had DM, 2.5% EM, 0.1% DA, 17.6% EA, and 8.5% CFD. Significant differences between urban and rural areas were found only for central fat distribution (X2: 22.61; p: 0.000) (Table 4).

**Table 4:** Differences in deficit and excess of muscular, adipose tissues and central fat distribution between urban and rural areas.

Body Composition	Prevalence (%)				
	Total	Urban	Rural	*X* ^2^	*p*
Deficit of muscular tissue	22.0	20.8	24.9	2.70	0.101
Excess of muscular tissue	2.5	2.3	3.0	0.65	0.420
Deficit of adipose tissue	0.1	0.2	0.0	0.82	0.365
Excess of adipose tissue	17.6	17.0	19.1	0.85	0.358
Central Fat Distribution	8.5	6.2	14.1	22.61	0.000

Table 5 shows the analysis of body composition in undernourished and overweight plus obesity groups. A total 53.0% of the undernourished children had muscular tissue deficit without significant urban-rural differences, and none of them showed deficit of adipose tissue. In the overweight plus obesity group, 62.8% of the children presented EA, 6.4% EM, 4.7% DM, and 22.8% CFD. Significant differences between rural and urban areas were recorded for deficit of muscular tissue and central fat distribution (with highest percentages in rural areas) (Table 5).

**Table 5:** Differences in body composition of undernourished and overweight plus obesity groups between urban and rural areas.

Groups	Prevalence (%)				
	Total	Urban	Rural	*X* ^2^	*p*
*Undernutrition*					
Deficit of muscular tissue	53.0	51.5	55.9	0.17	0.6785
Deficit of adipose tissue	0.0	0.0	0.0		
*Overweight+Obesity*					
Deficit of muscular tissue	4.7	2.9	9.0	5.22	0.0224
Excess of muscular tissue	6.4	7.2	4.5	0.37	0.5429
Excess of adipose tissue	62.8	61.7	65.2	0.32	0.5735
Central Fat Distribution	22.8	16.7	37.1	14.65	0.0001

## Discussion and Conclusions

The nutritional status of a community is an important indicator of its quality of life. This study provides new evidence of the nutritional situation of Argentinean children, and particularly of those living in Brandsen. Like many authors have observed in other Latin American populations, a low percentage of undernutrition (predominantly stunting) and high percentage of overweight and obesity were found [20]–[21]–[34]–[35]–[36]. Furthermore, similar results obtained in a previous work carried out in Brandsen lend support to the observed trend, which is characteristic of countries in nutritional transition [19]–[27]. Over several years, Popkin [37] has extensively analyzed the environmental factors associated to the obesity pandemic, and he has linked the changes in dietary patterns with those in physical activity to explain the nutrition transition that has been observed all over the world and mainly in developing countries.

Some authors have pointed out that cities are much more advanced along this nutritional transition than the countryside, since they have experienced the greatest increase of overweight and obesity [38]–[39]. In urban contexts the range of food choices is greater and prices are generally lower. On the other hand, urban employments often demand less physical exertion than rural ones, and a greater proportion of women work away from home and are too busy to shop for, prepare and cook healthy meals at home [15]. Nevertheless, we did not find urban-rural differences in overweight and obesity rates. According to Bogin [40], the migration of people, goods, and services along a path from urban to rural areas produces a “rural–urban continuum”, and rural families have greater access to food variety and quantity. Brandsen is a relatively small district, and the distance between rural and urban areas is relatively short, allowing uninterrupted connection between the countryside and the city. At the same time, in Argentina the mechanization of agricultural work, the improvement of public transport and the increased acquisition of motorized transport, have led to a significant reduction of physical activity [41]–[42].

No urban-rural differences were found in prevalence of underweight, stunting or wasting. The notion that urban children are less likely to suffer undernutrition than their rural counterparts is based on the idea that urbanization entails better access to food, services and employment opportunities [7]. However, in many cities of developing countries, not everyone is able to benefit equally and it is currently common to find great heterogeneity within a given urban context. According to Dufour and Piperata [43] cities contain more than one type of urban environment and the population that inhabits them is distributed according to SES. This statement suggests that cities are not uniform in terms of their health status. In agreement with these authors, the analysis of the socio-environmental survey showed that while access to public services like piped water, sewage system, waste collection, etc., and housing quality was significantly better in the urban area, a considerable number of city households lived under sanitary deficient conditions, lacked health insurance and had low socioeconomic level. The internal socio-environmental heterogeneity of Coronel Brandsen city could explain the lack of differences in the nutritional status of urban and rural children.

The assessment of body composition is important to evaluate nutrition and health status. Skinfold measurement is extremely common in epidemiological studies because they are inexpensive and simple to perform and allow the estimation of muscular and fat tissues from simple algorithms [44]. In this study, the body composition analysis showed that the amount of muscular and fat tissues of the arm was similar in all the children analyzed and independent of the environment of residence. Conversely, central fat distribution was more frequent in rural children. Although fat deposition patterns are known to be basically ruled by genetics [45], they are also associate to particular socio-cultural and environmental conditions [46]–[47]–[48]–[49]. The mechanism by which trunk fat deposition influences cardiovascular risk factors is not completely understood. However, central fat distribution is an important predictor of increase in plasma triglycerides, HDL cholesterol, systolic blood pressure, and left ventricular mass in children and adolescents [50]–[51]–[52]. Accordingly, these results may indicate that rural children are more prone to health problems.

On the other hand, body composition analysis in undernourished children indicated that about half of them had less muscular mass. Also, 63% of overweight and obese children presented excess of adipose tissue and 5% of them, reduction of muscular mass. This paradoxical coexistence was more evident in rural children, leading us to think that the diet eaten by these children was low in proteins and high in carbohydrates and lipids. Although we did not analyze food habits, Aguirre's [53]–[54] arguments support our interpretation. This author suggested that during the past decades in Argentina, a single pattern of consumption was replaced by two different types of diets: the “poor people's food and rich people's food”. The former is based on carbohydrates, fats and sugars and is cheaper, and the latter, which includes meat, dairy, fruits and vegetables rich in micronutrients, is more expensive [53]–[54]. Therefore, those children who consume diets with excessive amounts of carbohydrate and lipids but deficient in protein, are expected to present overweight or obesity and muscular deficit at the same time. In addition, the significant reduction of physical activity registered in all Argentinean children [55], and especially in those of rural population [42], could explain this paradoxical coexistence of obesity and reduced muscular mass.

The results obtained indicate the necessity of implementing policies and programs to improve socio-environmental conditions and allow better access to good nutrition for these children in order to protect their health and quality of life.
